# Dr. John Clark Cleary

**Published:** 1900-02

**Authors:** 


Dr. John Clark Cleary, U. of B., '98, of Port Chester, N. Y., died at his home January 14, 1900, of typhoid fever, aged 31 years. Dr. Cleary was a native of Clyde, N. Y., where he lived when he entered the University of Buffalo. He was a young man of promise, and his early death is deeply to be regretted.

